# Heart rate deceleration and acceleration capacities associated with circadian rhythm of blood pressure in essential hypertension

**DOI:** 10.1186/s12872-024-03933-9

**Published:** 2024-05-17

**Authors:** Jijing Wang, Jinyi Xu, Lihong Yang, You Zhang, Rui Wu, Wentao Wang, Chuanyu Gao

**Affiliations:** 1 Department of Cardiopulmonary Function, Zhengzhou University People’s Hospital, Henan Provincial People’s Hospital, No.7 Weiwu road, Jinshui District, Zhengzhou, Henan 450003 China; 2 Henan Institute of Cardiovascular Epidemiology, Zhengzhou, China; 3 Department of Cardiopulmonary Function, Central China Fuwai Hospital of Zhengzhou University, Henan Provincial People’s Hospital Heart Center, No. 1 Fuwai Road, Zhengzhou, Henan 451464 China; 4 Department of Cardiology, Central China Fuwai Hospital of Zhengzhou University, Henan Provincial People’s Hospital Heart Center, No. 1 Fuwai Road, Zhengzhou, Henan 451464 China

**Keywords:** Deceleration capacity, Heart rate variability, Circadian rhythm of blood pressure, Essential hypertension.

## Abstract

**Background:**

This study aimed to investigate the potential association between the circadian rhythm of blood pressure and deceleration capacity (DC)/acceleration capacity (AC) in patients with essential hypertension.

**Methods:**

This study included 318 patients with essential hypertension, whether or not they were being treated with anti-hypertensive drugs, who underwent 24-hour ambulatory blood pressure monitoring (ABPM). Patients were categorized into three groups based on the percentage of nocturnal systolic blood pressure (SBP) dipping: the dipper, non-dipper and reverse dipper groups. Baseline demographic characteristics, ambulatory blood pressure monitoring parameters, Holter recordings (including DC and AC), and echocardiographic parameters were collected.

**Results:**

In this study, the lowest DC values were observed in the reverse dipper group, followed by the non-dipper and dipper groups (6.46 ± 2.06 vs. 6.65 ± 1.95 vs. 8.07 ± 1.79 ms, *P* < .001). Additionally, the AC gradually decreased (-6.32 ± 2.02 vs. -6.55 ± 1.95 vs. -7.80 ± 1.73 ms, *P* < .001). There was a significant association between DC (*r* = .307, *P* < .001), AC (*r*=-.303, *P* < .001) and nocturnal SBP decline. Furthermore, DC (β = 0.785, *P* = .001) was positively associated with nocturnal SBP decline, whereas AC was negatively associated with nocturnal SBP (β = -0.753, *P* = .002). By multivariate logistic regression analysis, deceleration capacity [OR (95% CI): 0.705 (0.594–0.836), *p* < .001], and acceleration capacity [OR (95% CI): 1.357 (1.141–1.614), *p* = .001] were identified as independent risk factors for blood pressure nondipper status. The analysis of ROC curves revealed that the area under the curve for DC/AC in predicting the circadian rhythm of blood pressure was 0.711/0.697, with a sensitivity of 73.4%/65.1% and specificity of 66.7%/71.2%.

**Conclusions:**

Abnormal DC and AC density were correlated with a blunted decline in nighttime SBP, suggesting a potential association between the circadian rhythm of blood pressure in essential hypertension patients and autonomic nervous dysfunction.

**Supplementary Information:**

The online version contains supplementary material available at 10.1186/s12872-024-03933-9.

## Background

Hypertension is one of the most common cardiovascular diseases. In addition, it is a significant risk factor for the development of other cardiovascular diseases and mortality [1, 2]. Recently, according to international guidelines, there has been a growing recommendation for early, strict, and all-day blood pressure (BP) control. Consequently, there is an increasing utilization of out-of-office BP measurement methods such as home blood pressure monitoring and ambulatory blood pressure monitoring (ABPM) [3, 4]. These methods serve multiple purposes, including detecting nocturnal hypertension, evaluating the effectiveness of anti-hypertensive drugs, and analyzing the BP rhythm. ABPM can help reveal the circadian variation in BP. A normal circadian rhythm of BP is characterized by an increase upon waking in the morning and a decrease during sleep at night, which is defined as a dipper [5]. On the other hand, non-dipper refers to the absence or blunting of nighttime decreases in BP. The dipper is considered to indicate a normal physiological state, while the non-dipper is associated with an increased risk of target organ damage and cardiovascular events [6–8].

Previous studies have indicated that the autonomic nervous system (ANS) plays a role in regulating circadian BP variation [9]. These studies used heart rate variability indices as a measure of ANS function [10, 11]. By analyzing 24-hour Holter recordings, heart rate deceleration capacity and acceleration capacity can be calculated to assess sympathetic and vagus nerve modulation and predict cardiovascular and overall mortality [12–14]. However, there is a lack of research examining the association between deceleration and acceleration and circadian variation in BP. Therefore, our study aimed to investigate the impact of ANS function, as measured by deceleration and acceleration capacity, on the circadian rhythm of blood pressure in individuals with essential hypertension.

## Methods

### Study participants

This study retrospectively included a total of 318 essential hypertensive patients who were admitted to the People’s Hospital of Zhengzhou University. These patients underwent 24-hr ambulatory electrocardiography, transthoracic echocardiography, and ABPM from January 2020 to December 2022. Hypertension was defined as casual office systolic BP (SBP) ≥ 140 mm Hg and / or diastolic BP (DBP) ≥ 90 mm Hg, ABPM daytime SBP ≥ 135 mm Hg and / or DBP ≥ 85 mm Hg, ABPM nighttime SBP ≥ 120 mm Hg and / or DBP ≥ 70 mm Hg, ABPM 24-hr SBP ≥ 130 mm Hg and / or DBP ≥ 80 mm Hg, previously diagnosed hypertension, or currently using anti-hypertensive drugs [4].

Patients who met any of the following criteria were excluded: had (a) incomplete medical records; (b) severe arrhythmia, such as atrial fibrillation, ventricular tachycardia, ventricular fibrillation, type-II 2nd or 3rd-degree atrioventricular block, or sick sinus syndrome; or (c) a history of secondary hypertension, diabetes mellitus, renal insufficiency, acute coronary syndrome, severe valvular heart disease, obstructive sleep apnea syndrome, or malignant tumor.

This single-center retrospective study was approved by the Ethics Committee of The People’s Hospital of Zhengzhou University (2018 Ethics Review No. 24) before the operation. Because this was a retrospective observational study, the ethics committee of The People’s Hospital of Zhengzhou University waived the requirement for informed consent from eligible patients.

### ABPM recordings

Ambulatory blood pressure was monitored using a portable device (CB-1805-B, Vaso Medical Technology, Jiangsu, China). The left arm of hypertensive patients was selected for the placement of the cuff. As previously described [4], patients were instructed to maintain their daily routine during the monitoring period and to remain calm and maintain their body position when feeling the inflation of the cuff. BP were recorded according to fix intervals. BP was measured every 15 min during the day (6:00 a.m. to 10:00 p.m.) and every 30 min during the night (10:00 p.m. to 6:00 a.m.). Recordings with more than 70% valid BP measurements were considered reliable and were included in the final analysis [4].

The mean 24-hour, daytime, and nighttime SBP and DBP were recorded. Additionally, nocturnal SBP reductions were calculated as continuous variables using the following equation:

### Nocturnal SBP decline = [(Daytime mean SBP - nighttime

mean SBP)/daytime mean SBP] × 100%, as previously described [15].

According to the degree to which the nocturnal SBP decreased, the circadian BP pattern was classified as dipper (nocturnal SBP decline ≥ 10%), non-dipper (nocturnal SBP decline ≥ 0% and < 10%) or reverse dipper (nocturnal SBP decline < 0%).

### Holter recordings

A Holter monitor test was performed on each patient using a portable electrocardiogram device (CONTEC Medical System Ltd., Qinhuangdao, China). The test measures various indices, including average, fastest and slowest heart rate (HR), deceleration capacity, acceleration capacity, traditional heart rate variability (HRV) which included the standard deviation of normal-to-normal (NN) intervals (SDNN), the standard deviation average of NN intervals (SDANN), the root mean square successive difference of normal R-R intervals (RMSSD), and the percentage of the number of times that the difference between adjacent normal RR intervals > 50 ms occurred in the total number of NN intervals (PNN50). The SDNN and SDANN were considered to indicate vagal and sympathetic influences, respectively, while the RMSSD and PNN50 were regarded as indicators of parasympathetic nerve activity [16].

The heart rate deceleration and acceleration capacities were consider as novel HRV index, measured by the Holter system. The following calculation methods were used as previously described [17]: First, heart beat intervals were selected as decelerating anchors when > 1.00 but ≤ 1.05 of the preceding heart beat interval; heart beat intervals were selected as accelerating anchors when < 1.00 but ≥ 0.95 of the preceding heartbeat interval. Second, the heartbeat intervals around the decelerating and accelerating anchors were collected. Third, the above segments were aligned at the decelerating and accelerating points, and the signals of the segments were averaged to obtain the phase-rectified signal averaging signal X(i).

The following formula was used to quantify the deceleration capacity (DC) and acceleration capacity (AC): DC/AC = [X(0) + X(1) − X(− 1) − X(− 2)]/4.

When the DC is greater than 0, the vagus nerve activity is quantified, and when the AC is less than 0, the sympathetic nerve activity is quantified.

### Echocardiographic evaluation

In our study, all patients underwent transthoracic echocardiography (TTE) using a SonoS 5500 Ultrasound machine (Philips). The following parameters were measured by the M-mode technique: right atrial diameter (RAd) and left atrial diameter (LAd), left ventricular end-diastolic diameter (LVEDd), and left ventricular end-systolic diameter (LVESd). The Simpson’s biplane method was used to measure the left ventricular ejection fraction (LVEF).

### Statistical analysis

All the statistical analyses were performed using SPSS version 25.0 (IBM Corp., Armonk, NY). The Kolmogorov‒Smirnov test was used to assess the normality of the distribution. Normally distributed continuous variables are expressed as the mean ± SD, while non-Normally distributed parameters are presented as medians (interquartile ranges). Categorical data are presented as counts and percentages.

Depending on the nature of the data, differences among groups were compared using variance (ANOVA), Kruskal‒Wallis test, or χ2 test. Post hoc (Bonferroni) analysis was also conducted to further examine differences among groups. Pearson’s correlation coefficients were calculated to assess the correlation between continuous variables. Multiple linear regression analysis was carried out to evaluate the association between nocturnal SBP decline and DC/AC (including DC/AC, age, average HR, slowest HR, and SDNN). Multivariate logistic regression analysis was applied to determine independent risk factors for non-dipper BP pattern. Receiver operating characteristic (ROC) curve analysis was used to evaluate the predictive power of DC/AC for the circadian rhythm of blood pressure. All the statistical analyses were two-sided, and a P value < 0.05 was used to indicate statistical significance.

## Results

### Baseline characteristics and ambulatory blood pressure parameters among dipper, non-dipper, and reverse dipper groups

A total of 318 essential hypertensive patients were included in the study; 66 (20.75%) were classified as dippers, 140 (44.03%) as non-dippers, and 112 (35.22%) as reverse dippers. Compared to those of the dippers, the non-dippers and reverse dippers were more likely to be older (57.00 ± 10.48 vs. 60.10 ± 14.96 vs. 65.96 ± 13.97 years, *P* < .001). There were no significant differences in sex, body mass index (BMI), or the use of angiotensin-converting enzyme inhibitors/angiotensin receptor blockers, β-blockers, calcium channel blockers or diuretics (all *P* > .05).

### The nocturnal SBP (122.20 ± 7.46 vs. 130.21 ± 10.57 vs. 139.21 ± 13.89 mm hg) and

nocturnal DBP (73.26 ± 8.38 vs. 76.71 ± 9.85 vs. 77.04 ± 10.96 mm Hg) gradually increased in the dipper, non-dipper, and reverse dipper groups (all *P* < .05).

Conversely, the 24-hour DBP (81.73 ± 9.22 vs. 80.43 ± 9.59 vs. 76.18 ± 10.66 mm Hg), daytime SBP (141.82 ± 8.28 vs. 135.85 ± 10.35 vs. 131.70 ± 13.20 mm Hg), daytime DBP (84.62 ± 9.48 vs. 81.66 ± 9.75 vs. 75.84 ± 10.92 mm Hg) and nocturnal SBP decline (13.79 ± 3.28 vs. 4.15 ± 3.00 vs. -5.80 ± 4.37%) gradually decreased from the dipper, non-dipper, and reverse dipper groups (all *P* < .001). The 24-hour SBP was similar among the three groups (*P* = .111). The baseline characteristics and ambulatory blood pressure parameters of the dipper, non-dipper, and reverse dipper groups are summarized in Table 1.

**Table 1:** Baseline characteristics and ambulatory blood pressure parameters of the subjects

	Dipper	Non-dipper	Reverse dipping	χ^2^/F/H	*P*
N (%)	66(20.75)	140(44.03)	112(35.22)		
Age (years)	57.00 ± 10.48	60.10 ± 14.96^&^	65.96 ± 13.97*^#^	10.074	0.000
Female n (%)	29(43.94)	46(32.86)	47(41.96)	3.277	0.194
BMI (kg/m^2^)	25.46(24.04 ∼ 27.48)	25.70(23.60 ∼ 28.38)	25.74(23.88 ∼ 28.05)	0.411	0.814
**Medications n (%)**					
ACEI/ARB n (%)	21(31.82)	49(35.00)	33(29.46)	0.883	0.643
β-Blocke n (%)	12(18.19)	22(15.71)	18(16.07)	0.210	0.900
CCBs n (%)	32(48.49)	71(50.71)	59(52.68)	0.298	0.862
Diuretic n (%)	4(6.06)	8(5.71)	7(6.25)	0.033	0.984
**Ambulatory blood pressure parameters**					
24 h SBP (mmHg)	137.14 ± 8.03	134.51 ± 10.26	133.59 ± 13.14	2.216	0.111
24 h DBP(mmHg)	81.73 ± 9.22	80.43 ± 9.59	76.18 ± 10.66*^#^	8.426	0.000
Daytime SBP(mmHg)	141.82 ± 8.28	135.85 ± 10.35^&^	131.70 ± 13.20*^#^	17.369	0.000
Daytime DBP (mmHg)	84.62 ± 9.48	81.66 ± 9.75	75.84 ± 10.92*^#^	18.145	0.000
Night-time SBP(mmHg)	122.20 ± 7.46	130.21 ± 10.57^&^	139.21 ± 13.89*^#^	48.850	0.000
Night-time DBP (mmHg)	73.26 ± 8.38	76.71 ± 9.85^&^	77.04 ± 10.96*	3.433	0.034
Percentage of n-SBP decline (%)	13.79 ± 3.28	4.15 ± 3.00^&^	-5.80 ± 4.37*^#^	639.20	0.000

& compared with dipper group *P* < .05, * compared with dipper group *P* < .05, ^#^ compared with nondipper group *P* < .05, *BMI* Body mass index, *ACEI* Angiotensin-converting enzyme inhibitor, *ARB* Angiotensin-converting enzyme receptor blocker, *CCBs* Calcium channel blocker, *SBP* Systolic blood pressure, *DBP* Diastolic blood pressure

### Comparison of holter recording and echocardiographic parameters for dipper, non-dipper, and reverse dipper blood pressure measurements

**Fig. 1:**
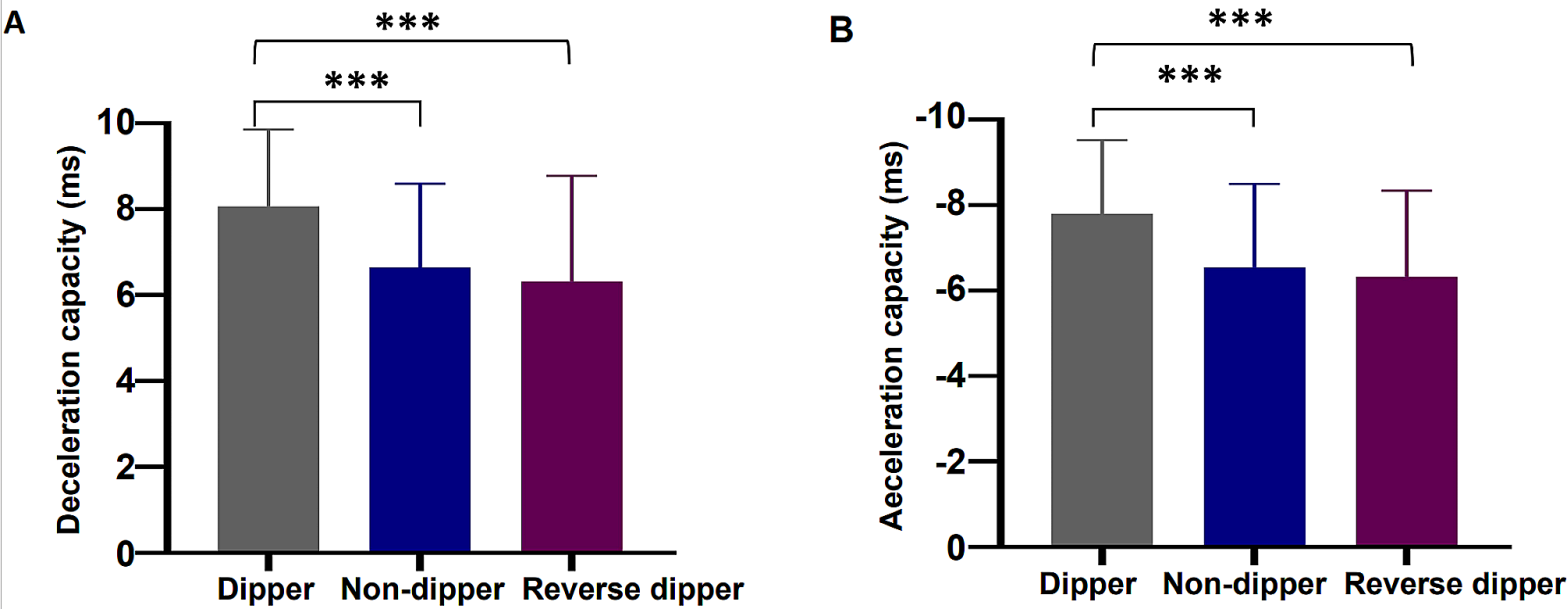
Comparison of DC/AC between dipper, non-dipper, and reverse dipper groups. (**a**) DC between dipper, non-dipper, and reverse dipper groups. (**b**) AC between dipper, non-dipper, and reverse dipper groups. *DC D*eceleration capacity, AC Acceleration capacity. *** indicates *P* < .001

As shown in Fig. 1, DC was lowest in the reverse dipper group, followed by the nondipper and dipper groups (8.07 ± 1.79 vs. 6.65 ± 1.95 vs. 6.46 ± 2.06 ms, *P* < .001) (Fig. 1A*)*. Conversely, the AC gradually decreased (-7.80 ± 1.73 vs. -6.55 ± 1.95 vs. -6.32 ± 2.02 ms, *P* < .001) (Fig. 1B).

The average HR (70.18 ± 7.71 vs. 75.16 ± 9.33 vs. 73.39 ± 8.98 bpm, *P* = .001) and the slowest HR (53.15 ± 4.80 vs. 55.02 ± 8.06 vs. 56.14 ± 7.39 bpm, *P* = .031) were greater in the non-dipper and reverse dipping groups, respectively, than in the dipper group.

The HRV indices SDNN (125.36 ± 21.22 vs. 114.99 ± 32.79 vs. 105.08 ± 30.01 ms, *P* < .001), SDANN [126.45 (107.25 ∼ 149.23) vs. 114.45 (91.65 ∼ 134.38) vs. 113.50 (84.30 ∼ 146.90) ms, *P* = .013], and PNN50 [6.15 (4.00 ∼ 10.13) vs. 4.00 (1.83 ∼ 8.08) vs. 4.40 (1.85 ∼ 6.70), *P* = .002] gradually decreased in the non-dipper and reverse dipper groups. However, the RMSSD did not significantly differ among the three groups.

In terms of echocardiographic parameters, the LVEF was lower in the non-dipper and reverse dipper groups than in the dipper group (65.77 ± 4.90 vs. 63.78 ± 5.21 vs. 64.08 ± 4.89 ms, *P* = .020). However, RAd, LAd, LVESd, and LVEDd did not significantly differ between dippers and non-dippers.

The Holter recording and echocardiographic parameters of the dipper, non-dipper, and reverse dipper groups are summarized in Table 2.

**Table 2:** Comparison of 24-hr ambulatory electrocardiographic and echocardiographic variables between dipper, non-dipper and reverse dipper groups

	Dipper(*n* = 66)	Non-dipper (*n* = 140)	Reverse dipper (*n* = 112)	F/H	*P*
**Deceleration and Acceleration capacities**					
Deceleration capacity (ms)	8.07 ± 1.79	6.65 ± 1.95^&^	6.46 ± 2.06*	15.153	0.000
Acceleration capacity (ms)	-7.80 ± 1.73	-6.55 ± 1.95^&^	-6.32 ± 2.02*	13.231	0.000
**Heart rate variability**					
Average HR (bpm)	70.18 ± 7.71	75.16 ± 9.33^&^	73.39 ± 8.98*	7.055	0.001
Slowest HR (bpm)	53.15 ± 4.80	55.02 ± 8.06	56.14 ± 7.39*	3.528	0.031
Fastest HR (bpm)	106.33 ± 12.90	111.59 ± 16.82	107.78 ± 109.16	2.849	0.059
SDNN (ms)	125.36 ± 21.22	114.99 ± 32.79^&^	105.08 ± 30.01*^#^	9.055	0.000
SDANN (ms)	126.45(107.25 ∼ 149.23)	114.45(91.65 ∼ 134.38)^&^	113.50(84.30 ∼ 146.90)*	8.745	0.013
RMSSD (ms)	58.00(30.80 ∼ 77.60)	49.60(31.93 ∼ 76.07)	48.05(31.33 ∼ 80.40)	1.035	0.596
PNN50 (%)	6.15(4.00 ∼ 10.13)	4.00(1.83 ∼ 8.08)^&^	4.40(1.85 ∼ 6.70)*	12.481	0.002
**Echocardiography**					
RAd (ms)	33.70 ± 3.98	33.52 ± 4.80	32.63 ± 3.59	1.834	0.161
LAd (ms)	35.55 ± 5.24	34.94 ± 5.40	34.67 ± 4.89	0.596	0.552
LVESd (ms)	29.24 ± 3.55	30.68 ± 3.92	29.77 ± 3.59	3.849	0.120
LVEDd (ms)	46.12 ± 3.85	47.22 ± 4.71	46.15 ± 4.61	2.250	0.107
LVEF(%)	65.77 ± 4.90	63.78 ± 5.21^&^	64.08 ± 4.89*	3.972	0.020

& compared with dipper group *P* < .05, * compared with dipper group *P* < .05, ^#^ compared with nondipper group *P* < .05, *BPM* Beat per min, *HR* Heart rate, *SDNN* Standard deviation of NN intervals, *SDANN* Standard deviation average of normal-to-normal intervals, *RMSSD* Root mean square of successive differences, *PNN50* The mean number of times in full course in which the change in successive normal sinus intervals exceeds 50 ms, *RAd* Right atrial diameter, *LAd* Left atrial diameter, *LVESd* Left ventricular end-systolic diameter, *LVEDd* Left ventricular end-diastolic diameter, *LVEF* Left ventricular ejection fraction

### Correlation analysis among ABPM parameters, heart rate variability, echocardiographic variables, heart rate deceleration capacity and acceleration capacity

**Fig. 2:**
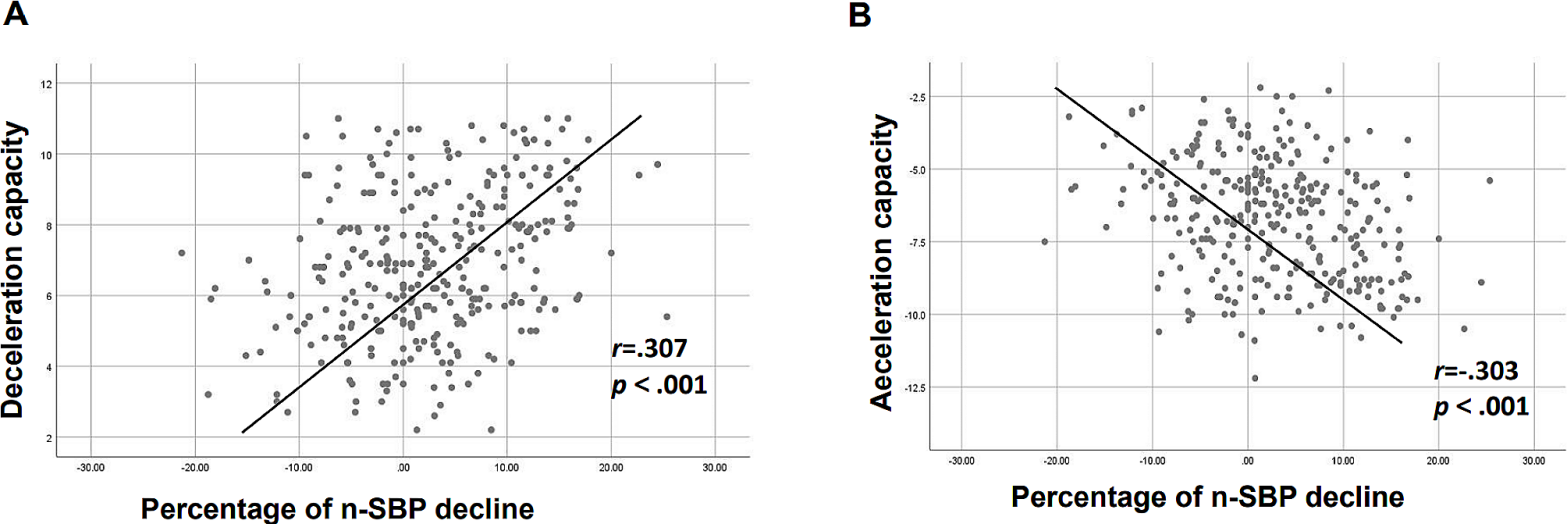
The relationship of **(a)** deceleration capacity, **(b)** acceleration capacity and percentage of n-SBP decline. *SBP* Systolic blood pressure

The results showed that age was negatively correlated with deceleration capacity (*r* = − .222, *p* < .001) and positively correlated with acceleration capacity (*r* = .255, *p* < .001). Additionally, 24-h DBP and daytime DBP were positively correlated with deceleration capacity and negatively correlated with acceleration capacity. Conversely, night-time SBP, similar to age, was negatively correlated with deceleration capacity and positively correlated with acceleration capacity (all *P* < .001). Furthermore, the percentage of n-SBP decrease was found to be positively correlated with deceleration capacity (*r* = .307, *p* < .001; Fig. 2A) and negatively correlated with acceleration capacity (*r* = − .303, *p* < .001; Fig. 2B).

Moreover, the SDNN showed a negative correlation with deceleration capacity (*r* = − .194, *p* = .021) and a positive correlation with acceleration capacity (*r* = .251, *p* = .003). The average HR and Slowest HR were also negatively correlated with deceleration capacity and positively correlated with acceleration capacity; correlation analysis results are presented in Table 3.

**Table 3:** Correlation analysis among 24-hr ambulatory blood pressure, echocardiographic recordings, acceleration capacity, and deceleration capacity

	Deceleration capacity		Acceleration capacity	
	*r*	*p*	*r*	*p*
Age	-0.222	0.000	0.255	0.000
24-h SBP	0.021	0.708	0.001	0.986
24-h DBP	0.187	0.000	-0.199	0.000
Daytime SBP	0.093	0.099	-0.070	0.216
Daytime DBP	0.214	0.000	-0.225	0.000
Night-time SBP	-0.186	0.001	0.200	0.000
Night-time DBP	0.073	0.192	-0.074	0.185
Percentage of n-SBP decline	0.307	0.000	-0.303	0.000
SDNN	0.389	0.000	-0.396	0.000
Average HR	-0.216	0.000	0.195	0.000
Slowest HR	-0.307	0.000	0.300	0.000
Fastest HR	-0.019	0.731	0.022	0.701
RAd	0.016	0.778	-0.013	0.811
LAd	0.023	0.645	-0.033	0.561
LVESd	-0.067	0.236	0.054	0.341
LVEDd	-0.028	0.642	0.017	0.757
LVEF	0.078	0.163	-0.076	0.178

*SBP* Systolic blood pressure, *DBP* Diastolic blood pressure, *SDNN* Standard deviation of NN intervals, *HR* Heart rate, *RAd* Right atrial diameter, *LAd* Left atrial diameter, *LVESd* Left ventricular end-systolic diameter, *LVEDd* Left ventricular end-diastolic diameter, *LVEF* Left ventricular ejection fraction

### Relationship between the percentage of nocturnal SBP decrease and DC/AC

We used multiple linear regressions to analyze the relationships between the percentage of nocturnal SBP decrease and DC (Table 4), AC (Table 5). Correlation analysis revealed that the models included DC/AC, age, average HR, slowest HR, SDNN. DC (β = 0.785, *P* = .001) and SDNN (β = 0.040, *P* = .024), which were positively associated with nocturnal SBP decline. On the other hand, AC (β = -0.753, *P* = .002) and age (β= -0.118, *P* < .001) were negatively associated with nocturnal SBP decrease.

**Table 4:** The relationships between percentage of nocturnal -SBP decline and deceleration capacity

	B	SE	β	t	*P*
Deceleration capacity	0.785	0.231	0.199	3.405	0.001
Age	-0.118	0.033	-0.208	-3.630	0.000
Average HR	-0.063	0.073	-0.070	-0.856	0.393
Slowest HR	0.044	0.097	0.040	0.455	0.649
SDNN	0.040	0.018	0.151	2.268	0.024

*B* the unstandardized beta coefficient, *SE* standard error, β the standardized coefficient, *HR*, heart rate, *SDNN* standard deviation of NN intervals

**Table 5:** The relationships between percentage of nocturnal -SBP decline and acceleration capacity

	B	SE	β	t	*P*
Acceleration capacity	-0.753	0.237	-0.187	-3.161	0.002
Age	-0.117	0.033	-0.206	-3.557	0.000
average HR	-0.067	0.073	-0.076	-0.922	0.357
Slowest HR	0.045	0.098	0.040	0.456	0.648
SDNN	0.040	0.018	0.153	2.281	0.023

*Note* B, the unstandardized beta coefficient; SE standard error; β, the standardized coefficient; HR, heart rate; SDNN, standard deviation of NN intervals

### Multivariate logistic regression analysis for the circadian blood pressure pattern

To determine the independent risk factors for non-dipper status, multivariate logistic regression analysis was performed in our study. According to the deceleration capacity model, deceleration capacity [OR (95% CI): 0.705 (0.594–0.836), *p* < .001], age [OR (95% CI): 1.039 (1.015–1.065), *p* = .002] and average HR (95% CI): 1.114 (1.052–1.181), *p* < .001] were identified as independent risk factors for BP non-dipper status (Table 6). Similarly, in the acceleration capacity model, we observed that acceleration capacity [OR (95% CI): 1.357 (1.141–1.614), *p* = .001], age [OR (95% CI): 1.039 (1.014–1.064), *p* = .002] and average HR [OR (95% CI): 1.114 (1.052–1.179), *p* < .001] were also independent risk factors for BP non-dipper status (Table 7).

**Table 6:** Multivariate logistic regression analysis for circadian BP pattern

	B	SE	Wald	*P*	OR(95%CI)
Deceleration capacity	-0.350	0.087	16.010	0.000	0.705(0.594–0.836)
Age	0.039	0.012	9.784	0.002	1.039(1.015–1.065)
average HR	0.108	0.030	13.435	0.000	1.114(1.052–1.181)
Slowest HR	-0.084	0.043	3.793	0.051	0.920(0.845–1.001)
SDNN	-0.009	0.006	2.095	0.148	0.991(0.979–1.003)

ORs for continuous variables = odds ratio for an increase in 1 unit. Values in bold indicate statistical significance (*p* < .05). *B* logistic coefficient, *CI* 95% confidence interval, *HR*, heart rate, *SDNN* standard deviation of RR intervals

**Table 7:** Multivariate logistic regression analysis for circadian BP pattern

	B	SE	Wald	*P*	OR(95%CI)
Acceleration capacity	0.305	0.088	11.906	0.001	1.357(1.141–1.614)
Age	0.038	0.012	9.505	0.002	1.039(1.014–1.064)
average HR	0.108	0.029	13.591	0.000	1.114(1.052–1.179)
Slowest HR	-0.079	0.042	3.503	0.061	0.924(0.850–1.004)
SDNN	-0.009	0.006	2.158	0.142	0.991(0.979–1.003)

ORs for continuous variables = odds ratio for an increase in 1 unit. Values in bold indicate statistical significance (*p* < .05). *B* logistic coefficient, *CI* 95% confidence interval, *HR*, heart rate, *SDNN* standard deviation of RR intervals

### ROC curve for predicting the circadian rhythm of blood pressure with DC and AC

**Fig. 3:**
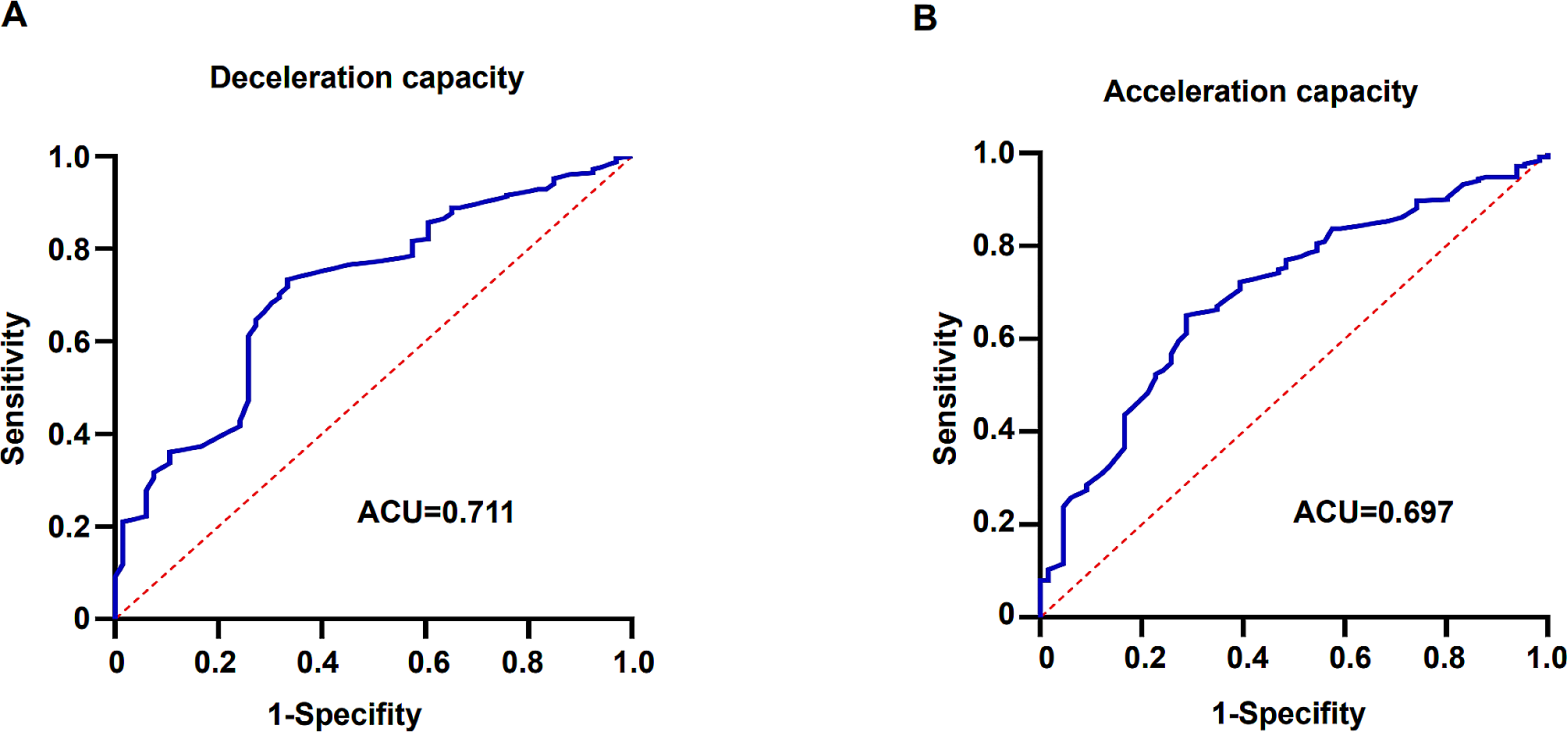
Receiver operating characteristic (ROC) curve for blood pressure circadian rhythms prediction with DC and AC. (**a**) ROC curve for prediction of blood pressure circadian rhythms with DC. (**b**) ROC curve for prediction of blood pressure circadian rhythms with AC. DC, deceleration capacity; AC, acceleration capacity; AUC, the area under the curve

ROC analysis was also conducted to evaluate the ability of DC and AC to predict the circadian rhythm of blood pressure (Fig. 3). Patients were divided into two groups based on dipping (nocturnal SBP decline ≥ 10%) or non-dipping (nocturnal SBP decline < 10%). The area under the curve (AUC) for DC in predicting the circadian rhythm of blood pressure was 0.711. With a cutoff of 7.75 ms, the sensitivity and specificity were 73.4% and 66.7%, respectively (Fig. 3A). Similarly, the AUC for AC in predicting the circadian rhythm of blood pressure was 0.697. With a cutoff of -7.05, the sensitivity and specificity of AC were 65.1% and 71.2%, respectively (Fig. 3B).

## Discussion

In a normal physiological state, BP is high during the day and low during the night. Dipper status, which is considered a normal physiological change, was defined as a decrease of more than 10% in nighttime BP compared to daytime BP. Moreover, non-dipper and reverse dipper statuses indicate a blunted nighttime drop in BP or even an increase in nighttime BP [5]. Previous studies have suggested that disturbed circadian BP variation is associated with damage to target organs and increased cardiovascular morbidity and mortality [6–8, 15, 18].

Various factors, including intrinsic and extrinsic factors, contribute to the disrupted circadian rhythm of BP. One of the most important factors for abnormal BP variation is an imbalance in the ANS [19, 20]. Impaired ANS function has been observed in non-dipper hypertensive patients. Cuspidi et al. [21] investigated the association between nocturnal BP patterns and sympathetic drive in essential hypertensive patients and reported a stepwise increase in sympathetic nerve activation from normotensive controls to extreme dipper, dipper, non-dipper, and reverse dipper hypertensive patients.

According to one study [22], non-dippers exhibit reduced nighttime decreases in norepinephrine and epinephrine levels, as well as heightened responsiveness of alpha 1-adrenergic receptors, in comparison to dippers. This finding suggested that sympathetic nervous system activity may be related to nighttime blood pressure fluctuations. Another study [20] revealed that an important regulatory mechanism for blood pressure in non-dippers is an increase in sympathetic tone over parasympathetic tone. However, it should be noted that the previous studies mentioned did not adequately quantify autonomic dynamics through the parameters of heart rate variability that were measured [23]. Cardiac function is regulated by the activity of the sympathetic and parasympathetic nervous systems. Heart rate variability (HRV) was used for the assessment of cardiac autonomic activity [24]. However, these HRV parameters measured in these previous studies cannot adequately quantify autonomic dynamics. Heart rate deceleration and acceleration capacities have recently emerged as novel indicators of ANS function, allowing for the quantification of sympathetic and vagus modulation [12]. A decrease in deceleration capacity has been identified as an independent predictor of mortality in post-myocardial infarction patients and a predictor of sudden cardiac death in heart failure patients [12, 14, 25, 26]. Acceleration capacity has been shown to be a predictor of heart failure exacerbation in dilated cardiomyopathy patients [13].

Despite these findings, studies investigating heart rate acceleration and deceleration in hypertensive patients are still limited. XD Wang et al. examined the heart rate (HR) DC and deceleration running (DRs) in patients with T2DM, with or without essential hypertension, and observed that patients with T2DM, with or.

without hypertension had significantly lower DC and DRs than did the healthy controls [27]. LY Yan et al. reported that the absolute values of AC and DC were lower in non-dipper hypertension patients than in dipper hypertension patients. However, DC and AC were not identified as independent risk factors for BP non-dipper status [17].

In our study, we evaluated autonomic nervous function using DC and AC in patients with essential hypertension who had different BP dipping patterns. We found that DC density was significantly lower in non-dippers and reverse dippers than in dippers. However, the AC gradually increased from dippers to non-dippers to reverse dippers. Notably, we observed a significant association between decreased DC and elevated AC with a decrease in nocturnal SBP in patients with essential hypertension. Additionally, we identified DC and AC as independent risk factors for non-dipper BP. These findings suggest that blunted nocturnal SBP decline is linked to autonomic nervous dysfunction, involving both sympathetic nerve activation and vagus nerve reduction.

Our study revealed that age was an independent risk factor for non-dipper BP. Age was greater in the non-dipper and reverse dipper groups than in the dipper group, and DC decreased with increasing age [28]. Autonomic nervous dysfunction is an independent predictor of hypertension and is increasingly associated with age-related physiological alterations [29]. With increasing age, several components of the autonomic nervous system undergo changes, including baroreceptor reflex function, plasma levels of norepinephrine, β-adrenoceptor sensitivity, and postreceptor signaling [30, 31]. As a result, age-related changes in ANS function may lead to a decrease in nocturnal blood pressure and impairment of ANS activity.

The average and slowest heart rates were significantly greater in the non-dipper and reverse dipping groups. Additionally, the average HR was identified as an independent risk factor for BP non-dipper status. As previously mentioned, the elevated average and slowest heart rates observed in non-dippers compared to dippers may be attributed to impaired sympathetic and vagus activity. Notably, baseline clinical heart rate and heart rate changes during the first few months of follow-up are independent predictors of the development of sustained hypertension in young persons screened for stage 1 hypertension [32].

The SDNN, which is an index of traditional HRV, was measured by Holter monitoring, which reflects the balance between the sympathetic nervous system and vagus nervous system in controlling heart rate [33]. The study showed that the SDNN was significantly lower in the non-dipper and reverse dipping groups than in the dipper group. This finding suggested that patients with abnormal blood pressure fluctuations have an impaired sympathovagus balance. However, it is important to note that this assessment combines the evaluation of sympathetic nerves with that of vagus nerves and can provide qualitative, not quantitative, assessments only.

The impact of anti-hypertensive drugs on the circadian rhythm of blood pressure is still unclear. Although our study did not find a direct alteration in blood pressure circadian rhythm with anti-hypertensive drug usage, other studies indicate that certain medications may be beneficial in addressing this issue. For example, a PRISMA subgroup analysis conducted in 2014 revealed that telmisartan treatment was linked to a greater likelihood of normalizing abnormal blood pressure circadian rhythm compared to ramipril, as well as a notable decrease in morning blood pressure [34].

It is currently understood that β-blockers notably reduce sympathetic nerve activity, potentially through a increase in vagus nerve activity, while the effects of.

Angiotensin-converting enzyme inhibitor(ACEI)/Angiotensin-converting enzyme receptor blocker(ARB), calcium channel blockers (CCBs), and diuretics on the sympathetic nervous system are still debated [35]. Some studies indicate that ACEI/ARB can ameliorate autonomic dysfunction in patients with diabetes. However, there is no evidence that these drugs specifically influence the deceleration capacity or deceleration runs [27, 36]. There is no conclusive evidence that the use of these anti-hypertensive drugs in hypertensive patients directly impacts deceleration or acceleration capacities.

The night-time and day-time periods were defined as fixed intervals not actual wake/sleep period. Day-time (periods of wakefulness, 6:00 a.m. to 10:00 p.m.) and night-time periods of sleep, 10:00 p.m. to 6:00 a.m.). The patients in this study had relatively regular day and night life, and did not include shift workers, so the blood pressure were recorded according to fix intervals [4].

## Conclusions

In conclusion, our study established significant associations between decreased DC and increased AC and abnormal BP circadian rhythms in individuals with essential hypertension. Additionally, we identified DC, AC, age, and average HR as independent risk factors for BP nondipper status. Therefore, DC and AC could serve as potential indices for predicting the circadian rhythm of blood pressure. Furthermore, our findings highlight the presence of autonomic nervous dysfunction in the circadian rhythm of blood pressure in essential hypertension patients, involving both sympathetic nerve activation and vagus nerve reduction.

## Limitations

This study has several limitations. First, it had a single-center retrospective design, and additional prospective studies are needed to investigate the association between deceleration and acceleration and BP variation. Second, the study did not include a normotensive group. We plan to include a more comprehensive range of cases in subsequent studies. Third, the area under the curve (AUC) values of the receiver operating characteristic (ROC) curve were not too high, but the relationship between DC/AC and BP circadian rhythms was not affected. In addition, the use of anti-hypertensive drugs was not excluded in the study, some patients may have been taking medications like ACEIs, ARBs, or β-blockers that could affect autonomic nervous system ANS function and further research is needed to explore this area. In addition, hormone replacement therapy was not recorded during the study inclusion period. Because patients did not individually record their bedtime or wake time, we used fixed intervals and not actual wake/sleep period definitions. Finally, The ambulatory BP monitor used in this study has not been clinically validated according to an international validation protocol.Thus, its accuracy cannot be ascertained.

### Electronic supplementary material

Below is the link to the electronic supplementary material.

Supplementary Material 1

## Data Availability

We collected the data for this study are available from the People’s Hospital of Zhengzhou university, but restrictions apply to the availability of these data, which were used under license for the current study, and so are not publicly available. The datasets used and/or analyzed during this study are available from the corresponding author on reasonable request. Requests to access these datasets should be directed to author, gaocy68020@163.com.

## Abbreviations

DC: Deceleration capacity
AC: Acceleration capacity
SBP: Systolic blood pressure
DBP: Diastolic blood pressure
BP: Blood pressure
ABPM: Ambulatory blood pressure monitoring
ANS: Autonomic nervous system
HRV: Heart rate variability
SDNN: Standard deviation of normal R-R intervals
SDANN: Standard deviation average of normal-to-normal intervals
RMSSD: Root mean square successive difference of normal R-R intervals
PNN50: Percent of the number of times that the difference between adjacent normal RR intervals > 50 ms in the total number of NN intervals
TTE: Transthoracic echocardiography
RAd: Right atrial diameter
LAd: Left atrial diameter
LVEDd: Left ventricular end-diastolic diameter
LVESd: Left ventricular end-systolic diameter
LVEF: Left ventricular ejection fraction
ROC: Receiver operating characteristic
BMI: Body mass index
AUC: Areas under the curve
