# Supplementary material for: Heart rate deceleration and acceleration capacities associated with circadian rhythm of blood pressure in essential hypertension
Source: BMC Cardiovasc Disord. 2024 May 17;24:257. doi: 10.1186/s12872-024-03933-9 (PMC11100176; doi:10.1186/s12872-024-03933-9)
Supplement: Supplementary file 1 — Supplementary Material 1 [file 12872_2024_3933_MOESM1_ESM.docx]

**Supplemental Table 1** Gender, Age and BMI were used as covariates to correct the DC and AC values between different groups of Circadian rhythm of BP

| Dependent variable | Covariates |  | F | Significance | Partial Eta squared |
| --- | --- | --- | --- | --- | --- |
| Deceleration Capicity | Gender | Gender | 0.016 | 0.901 | 0.000 |
|  |  | Circadian rhythm of BP | 15.097 | ＜0.001 | 0.088 |
|  | Age | Age | 10.502 | ＜0.001 | 0.032 |
|  |  | Circadian rhythm of BP | 11.795 | ＜0.001 | 0.070 |
|  | BMI | BMI | 3.621 | 0.058 | 0.011 |
|  |  | Circadian rhythm of BP | 15.524 | ＜0.001 | 0.090 |
| Acceleration Capacity | Gender | Gender | 0.103 | 0.748 | 0.000 |
|  |  | Circadian rhythm of BP | 13.204 | ＜0.001 | 0.078 |
|  | Age | Age | 15.369 | ＜0.001 | 0.047 |
|  |  | Circadian rhythm of BP | 9.907 | ＜0.001 | 0.059 |
|  | BMI | BMI | 4.932 | 0.027 | 0.015 |
|  |  | Circadian rhythm of BP | 13.682 | ＜0.001 | 0.080 |

**Supplemental Table 2** Comparison of 24-hr ambulatory electrocardiographic and echocardiographic variables between different gender in three groups.

|  | Dipper  （n=66） | | Non-dipper  （n=140） | | Reverse dipper  （n=112） | |
| --- | --- | --- | --- | --- | --- | --- |
|  | Male  n=41 | Female  n=25 | Male  n=94 | Female  n=46 | Female  n=65 | Female  n=47 |
| DC (ms) | 8.07±1.90 | 8.08±1.58 | 6.80±2.07 | 6.46±1.79 | 6.29±2.02 | 6.45±3.05 |
| AC (ms) | -7.85±1.82 | -7.84±1.75 | -6.66±2.03 | -6.50±1.75 | -6.17±2.0 | -6.62±2.14 |
| Average HR (bpm) | 70.88±8.40 | 69.04±6.42 | 76.87±9.58 | 71.67±7.78* | 74.29±8.76 | 72.15±9.23 |
| Slowest HR (bpm) | 53.10±5.21 | 53.24±4.16 | 55.95±7.92* | 53.13±8.10 | 56.82±7.41 | 55.21±7.34 |
| Fastest HR (bpm) | 106.85±13.61 | 105.48±11.87 | 113.62±15.91 | 107.43±18.00 | 108.65±15.84 | 106.57±20.99 |
| SDNN (ms) | 124.17±22.04 | 127.28±20.08 | 111.48±32.21 | 122.33±33.06 | 105.68±30.69 | 104.45±29.24 |
| SDANN (ms) | 126.00(113.00~146.50) | 138.00(100.00~160.50) | 114.50(95.0~134.50) | 115.50(83.0~137.50) | 117.0(83.00~152.50) | 108.0(84.00~138.00) |
| RMSSD (ms) | 51.00(28.00~76.50) | 66.00(43.50~90.00) | 43.50(28.75~65.50) | 58.50(42.75~99.25)* | 45.0(30.00~87.00) | 50.0(37.0~74.00) |
| PNN50 (%) | 5.00(2.50~10.00) | 7.00(4.00~11.00) | 4.00(2.00~8.00) | 5.00(3.00~9.00) | 4.00(1.50~6.00) | 5.00(2.00~8.00) |
| RAd (ms) | 34.49±4.31 | 32.40±3.03**^&^** | 33.95±4.36 | 32.91±5.67 | 33.22±3.28 | 31.83±3.87 |
| LAd (ms) | 35.34±5.60 | 35.88±4.69 | 35.46±5.14 | 33.8±5.82 | 35.32±4.8 | 33.77±4.91 |
| LVESd (ms) | 29.58±3.69 | 28.68±3.29 | 31.26±4.06 | 29.31±3.31* | 30.48±3.8 | 28.79±3.07**^#^** |
| LVEDd (ms) | 46.51±4.18 | 45.48±3.23 | 47.95±4.69 | 45.64±4.28* | 47.09±4.61 | 44.85±4.33**^#^** |
| LVEF(%) | 65.54±5.11 | 66.16±4.61 | 63.33±5.36 | 64.73±4.97 | 63.8±4.75 | 64.47±3.62 |

**^&^** In dipper group, compared with Male group *P* <0.05, * In non-dipper group, compared with Male group *P* <0.05, **^#^**In reverse-dipper group, compared with Male group *P* <0.05, *HR* Heart rate, *SDNN* Standard deviation of NN intervals, *SDANN* standard deviation of normal-to-normal intervals, *RMSSD* Root mean square of successive differences, *PNN50* The mean number of times in full course in which the change in successive normal sinus intervals exceeds 50 ms, *RAd* Right atrial diameter, *LAd* Left atrial diameter, *LVESd* Left ventricular end-systolic diameter, *LVEDd* Left ventricular end-diastolic diameter, *LVEF* Left ventricular ejection fraction.
